# Construction and Adsorption Performance Study of GO-CNT/Activated Carbon Composites for High Efficient Adsorption of Pollutants in Wastewater

**DOI:** 10.3390/polym14224951

**Published:** 2022-11-16

**Authors:** Hao Li, Tiehu Li, Tongyu Zhang, Jiajia Zhu, Weibin Deng, Delong He

**Affiliations:** 1 School of Materials Science and Engineering, Northwestern Polytechnical University, Xi’an 710072, China; 2 Laboratoire de Mécanique Paris-Saclay, Université Paris-Saclay, CentraleSupélec, ENS Paris-Saclay, CNRS, 91190 Gif-sur-Yvette, France

**Keywords:** kinetic study, graphene oxide, CNT, activated carbon, composites, adsorption

## Abstract

Based on the increasing application requirements for the efficient adsorption of wastewater pollutants, graphene oxide-carbon nanotube/activated carbon (GO-CNT/AC) composites are constructed from the optimal microstructure matching of GO, CNTs, and AC materials by solution impregnation and freeze-drying methods. Three-dimensional structures with nano-micro hierarchical pores are established, with GO and CNTs uniformly dispersed on the AC surface, effectively restrain the agglomeration. The added CNTs played a “spring” role, supporting the gap between the GO sheets and AC matrix. Meanwhile, stable links are formed between GO, CNTs, and AC, realizing the synergistic matching of the microstructure, which provides abundant active absorption sites beneficial for improving the adsorption performance. The influences of the CNT contents, adsorbent amounts, methylene blue (MB) concentrations, and pH values on the adsorption property of GO-CNT/AC composites are systematically investigated. The results show that when the pH value of the MB solution is 13, the CNT concentration is 3 mg/mL and the MB concentration is 200 mg/L, the adsorption property of the composite is the best, with an adsorption capacity of 190.8 mg/g and a removal percentage of 95.4%. Compared with the raw AC, the adsorption capacity and removal percentage of the composites are increased by 73.9% and 72.8%, respectively. The GO-CNT/AC composites exhibit excellent cyclic adsorption performance, with a cyclic stability of 91.8% after six rounds of adsorption–desorption cycles. The kinetic analysis shows that the adsorption process conforms to the PSO kinetic model. By fitting of the IPD model, the adsorption mechanisms of the GO-CNT/AC composites are divided into two adsorption stages and described respectively. This study provides a new way to achieve highly efficient adsorption of pollutants in wastewater.

## 1. Introduction

With the development of modern industry, an increasing number of industrial pollutants, especially industrial dyes and heavy metal ions, enter water bodies causing continuous and serious harm to the human living environment and health. How to remove pollutants from wastewater has become a key problem to be solved [1,2]. At present, various methods including adsorption, degradation, and flocculation, etc., are used to remove harmful substances from wastewater. Arif et al. [3,4] designed and further compounded different metal nanoparticles with microgels and obtained novel Cu-hybrid microgels and Co-hybrid microgels, which were used to degrade 4-nitrophenol (4NP), methyl blue (MB), and methyl orange (MO) in aqueous medium, with excellent degradation effects. They also reported the complete process of other types of hybrid microgels in the removal of toxic pollutants [5,6,7], which showed great application potential in the field of catalytic degradation of organic dyes and toxic chemicals. On the other hand, the methods of using absorbent materials with good adsorption performance, such as activated carbon (AC), zeolite and porous nanomaterials, to adsorb and remove pollutants in wastewater have also been widely adopted [8,9]. However, the existing adsorbent still has shortcomings, such as relatively low adsorption properties and recycling performance [10,11]. Therefore, explorations on developing a new adsorbent with excellent adsorption performance and good recycling adsorption stability have attracted extensive attention of the researchers.

AC is an effective broad-spectrum dye adsorbent [12], which has good adsorption capacity for dyes, waste gas, and heavy metal ions, and has the advantages of green environmental protection, low cost, and convenience for use. Therefore, AC materials are widely used in the treatment of dangerous goods, waste gas and water purification. Arami et al. [13] prepared KOH-activated AC materials using coal tar pitch and pulverized coal as precursors. The BET test shows that this material has developed a microporous structure, with a specific surface area of 1044 m^2^/g and an adsorption capacity of 7.4 mol/kg for carbon dioxide. Naushad et al. [14] used arginine to modify raw AC. The maximum adsorption amount of methylene blue (MB) was 219.9 mg/g, and its stability reached 75% after four cycles of adsorption experiments. Liu et al. [15] used Fe_3_O_4_ nanoparticle functionalized AC to prepare Fe_3_O_4_/AC composites with developed structures. Rhodamine B and methyl orange were used to characterize the adsorption performance. The Langmuir theoretical maximum adsorption capacity was 182.5 mg/g and 150.4 mg/g by electrostatic attraction and meso-porous filling respectively. Boruban et al. [16] compounded copper oxide nanoparticles with AC by the impregnation method, and the adsorption capacity of AC on carbon dioxide increased by 70% due to copper oxide nanoparticles loading. Ren et al. [17] modified Fe(NO_3_)_3_ by immersion calcination and prepared modified Fe-AC, which can effectively remove ammonia nitrogen from low concentration wastewater. However, the adsorption of AC materials mainly depends on the filling effect of its microporous or mesoporous structure. This kind of adsorption is difficult to meet the requirements of rapid adsorption and cyclic adsorption, so it needs to be further modified to meet the increasing complex application environments.

As a material with a unique two-dimensional structure, graphene oxide (GO) has excellent adsorption properties, which is widely used in dye pollution treatment [18,19,20], waste gas treatment [21,22], heavy metal pollution treatment [23], etc. Wang et al. [24] prepared a new CoS_2_/GO nanocomposite, which has good adsorption performance for Hg, with a maximum adsorption capacity of 1267 mg/g. The effective adsorption rate reaches 99.9%. Su et al. [25] prepared a new GO/CMC composite adsorbent by a solution blending method, and the maximum adsorption capacity to UDMH was 3.24 mg/g. Natesan et al. [26] added GO-CuO into cellulose acetate and polyethersulfone blend polymers through a phase inversion process to prepare thin film nanocomposite (TFN) membranes for dye pollutant removal. Due to the strong interaction between cationic dye molecules on the adsorbent surface, the adsorption performance of TFN membranes and cationic dye molecules is greatly improved. Zhang et al. [27] prepared MIL-100@GO composites, which showed excellent adsorption properties for methyl orange, Congo red, methylene blue, and acid chrome blue K. Deng et al. [28] prepared an amino-modified zirconium-based MOF (UiO-66-NH_2_) onto GO to prepare GO@UiO-66-NH_2_ composites used to adsorb Sb in mine wastewater, and the adsorption efficiency reached 93.79%.

Carbon nanotubes (CNTs) have a hollow tubular structure and very high specific surface area, which can provide broad adsorption sites for adsorbates, revealing excellent electrostatic adsorption performance for ionic dyes with positive electricity [29]. Fu et al. [30] obtained oxidized MWCNTs (OMWCNTs) by introducing hydroxyl (-OH) and carboxyl (-COOH) functional groups on the surface of MWCNTs to remove Pb(II) from wastewater. The adsorption of Pb(II) by OMWCNTs can reach equilibrium within 10 min, and the maximum adsorption capacity is 558.66 mg/g. Wang et al. [31] synthesized multiwalled magnetic CNTs by oxidation of reinforces and modification of magnetic nanoparticles, which had good selective adsorption performance for Pb(II), and the maximum adsorption capacity was 215.05 mg/g. Yu et al. [32] used acrylic acid, acrylamide, and hydrophilic CNTs to prepare hydrophilic multiwall CNT hydrogels for the adsorption of water-soluble cationic dyes. The MWCNT hydrogel has good adsorption properties for saffron T, crystal violet, and malachite green. Under the optimal conditions, the adsorption capacity of the hydrogel for saffron T was 913.64 mg/g, and the adsorption rate was 91.36%; the adsorption capacity of crystal violet was 959.37 mg/g, and the adsorption rate was 95.94%; the adsorption capacity of malachite green was 821.27 mg/g, and the adsorption rate was 82.13%.

At present, GO and CNTs are highly efficient and widely adopted in adsorbing and removing pollutants from wastewater; however, due to their small size, they are easy to be discharged into the water environment and difficult to separate from aqueous solutions, resulting in secondary pollution. In addition, these nanoscale additives are easy to agglomerate in the cyclic adsorption–desorption processes, which leads to the decline or even failure of adsorption properties. Therefore, it is necessary to explore a new method to construct GO and CNT composites with excellent dispersion and high cycle stability. For this purpose, GO-CNT/AC composites were designed and further synthesized via solution impregnation method and freeze drying method, to achieve good adsorption property and cycle stability for highly efficient removal of pollutants in wastewater. For this novel composite, GO and CNTs were uniformly dispersed on the AC surface, to effectively restrain the agglomeration. The addition of CNTs played a “spring” role, supporting the gap between the GO sheets and AC. Meanwhile, stable links are formed between GO, CNTs, and AC, realizing the synergistic matching of the microstructure, which provides abundant active absorption sites beneficial for improving the adsorption performance. To investigate the adsorption performance of the composites, MB was chosen as the simulated pollutant. Finally, the GO-CNT/AC composites obtain the best absorption properties, with an adsorption capacity of 190.8 mg/g and a removal percentage of 95.4% for MB solution, as well as exhibiting excellent cyclic adsorption performance, with a cyclic stability of 91.8% after six rounds of adsorption–desorption cycles.

## 2. Materials and Methods

### 2.1. Materials

Activated carbon used as the matrix of the GO-CNT/AC composites was purchased from Tianjin Damao Chemical Reagent Factory (Tianjin, China); flake graphite used to prepare GO was purchased from Nanjing XFNANO Materials Tech Co., Ltd. (Nanjing, China), and CNTs used to prepare GO-CNT/AC composites were also purchased from Nanjing XFNANO Materials Tech Co., Ltd. (Nanjing, China).

### 2.2. Preparation of GO

GO was prepared by a modified Hummers’ method in this study. First, 1 g of flake graphite and 1 g of NaNO_3_ were put into a beaker with 50 mL of concentrated H_2_SO_4_ and stirred for 30 min, and 6 g of KMnO4 was slowly added to the beaker, with the reaction temperature set below 20 °C for 1 h. Then, 80 mL of water was added at a constant temperature of 95 °C and held for 0.5 h. After that, when the solution decreased to room temperature, 200 mL of deionized water and 6 mL of H_2_O_2_ were added and stirred for 15 min, and then, the solution was washed and filtered with 5% HCl until the pH value reached 7. Finally, after drying in a freeze dryer, the GO sample was obtained.

### 2.3. Acidizing Treatment of CNTs

To improve the dispersion of CNTs and make them more evenly distributed in the solution, CNTs were acidified to increase the number of functional groups. About 1 g of CNTs was put in a 120 mL mixed solution of concentrated H_2_SO_4_ and concentrated HNO_3_. After stirring and ultrasonic treatment for 1 h, the acidified CNT solution was further washed and filtrated. Then, the freeze-drying process was adopted to obtain acidified CNT powder.

### 2.4. Preparation of GO-CNT/AC Composites

The GO-CNT/AC composites were prepared by the solution impregnation method. The pretreated CNTs and GO were put into deionized water with 200 mg of sodium dodecyl sulfate (SDS), and treated with a cell ultrasonic pulverizer for 50 min to prepare a GO-CNT solution with a GO concentration of 2 mg/mL, the CNT concentration varied from 0 mg/mL to 5 mg/mL. Then, 3 g of AC was immersed into 50 mL of GO/CNT solution for 24 h. After filtration, the samples were freeze dried at −35 °C for 48 h to obtain GO-CNT/AC composites. In particular, when CNT concentration of 0 mg/mL was applied, the GO/AC composite was obtained for comparison.

### 2.5. Characterization

The morphology of the GO-CNT/AC composites was analyzed by scanning electron microscopy (SEM, FEI Helios, Hillsboro, TX, USA). The crystal structure was analyzed by X-ray diffractometer (XRD, Nalytical X’Per PRO, Almelo, The Netherlands) with a copper target and wavelength of 0.154 nm. The microstructure and defects were measured by a Raman spectrometer (Raman, Alpha300R, Ulm, Germany). The components of the composite were determined by X-ray photoelectron spectrometer (XPS, Thermo Scientific K-Alpha, Waltham, MA, USA). The thermal stability was investigated by a thermal gravimetric analyzer (TGA, NETZSCH TG 209F3, Selb, Germany). The N_2_ adsorption–desorption isotherms were analyzed by a surface area and porosimetry system (BET, Micromeritics, ASAP2460, Norcross, GA, USA). The absorbance of the adsorbate solution was measured by a double-beam ultraviolet visible photometer (UV-Vis, TU-1810, Beijing, China).

The adsorption performance of the GO-CNT/AC composites was tested using MB as the target pollutant. To carry out the measurement, the composites were immersed into the MB solution with different initial concentrations; the MB solution concentrations at different adsorption times were recorded. Then, the removal percentage R (%) and the adsorption capacity Q_t_ (mg/g) of the GO-CNT/AC composites for MB can be calculated using the following formulas.

(1) $$R=(C_{0}-C_{t})/C_{0}\cdot 100\%$$

(2) $$Q_{t}=(C_{0}-C_{t})\cdot V/m$$

where C_0_ is the initial concentration of the MB solution (mg/L), C_t_ is the concentration of MB solution at time t (mg/L), V is the volume of the MB solution (L), and m is the mass of the adsorbent (mg).

To carry out the desorption process, the samples that experienced MB adsorption were immersed in 0.1 mol/L of HCl for 6 h. Then, the samples were washed with deionized water and freeze dried to obtain desorbed composites.

## 3. Results and Discussion

### 3.1. Morphology and Structure Analysis of the GO-CNT/AC Composites

**Figure 1:**
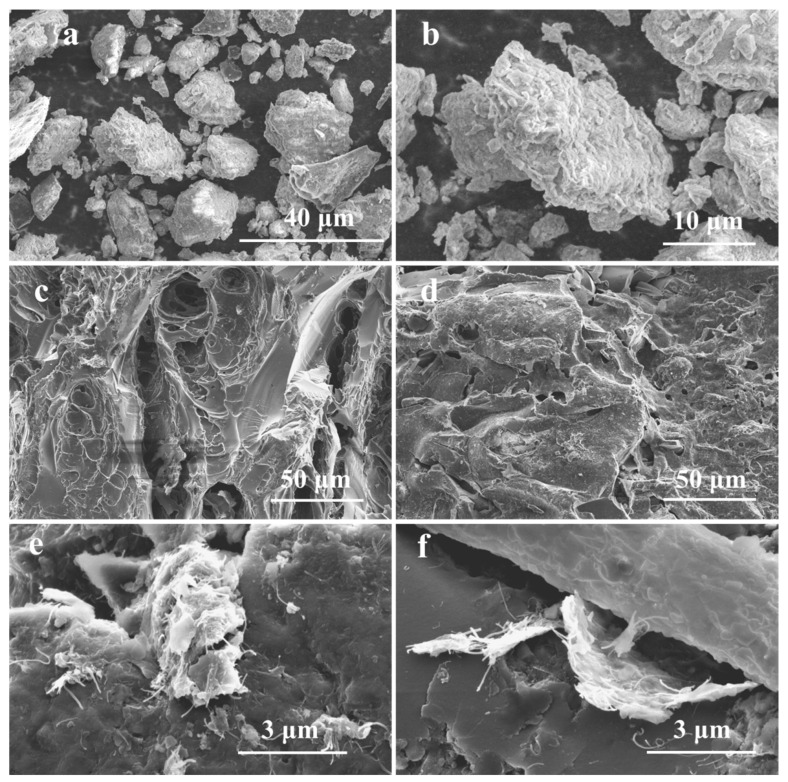
SEM images of AC under different magnifications (**a**,**b**), GO/AC composites (**c**), GO-CNT/AC composites (**d**), high magnification images of GO-CNT/AC composites (**e**,**f**).

The morphology and microstructure of the AC, GO/AC, and GO-CNT/AC composites are shown in Figure 1. The SEM images of AC in Figure 1a reveals the existence of two particle size distribution ranges, which are fine particles with a size range of 5–10 μm and large particles with a size range of 20–30 μm. The high magnification image in Figure 1b reveals the loose porous structures on the surface of AC particles, which is conducive to further combination with GO and CNTs. During the preparation process of the GO-CNT/AC composites, AC particles are interconnected with CNTs and GO sheets to form large bulk composites with three-dimensional structure, as shown in Figure 1c,d. The nano-micro hierarchical porous structures are thus constructed, providing extremely high surface areas and abundant channels for the diffusion and deposition of adsorbates. After the addition of CNTs, the image of the matrix surface of GO-CNT/AC composite shows the uniform distribution of CNTs and GO. Meanwhile, because the CNTs and GO sheets experienced ultrasonic dispersion in the preparation process, forming a three-dimensional structure that intersected with each other, this morphology is maintained in the subsequent structure of the composites. Figure 1e,f are the high magnification images of Figure 1d, which clearly show the combination state of GO sheets, CNTs, and AC matrix. The SEM images reveal that the composite has a three-dimensional structure with nano-micro hierarchical pores, which provides a large specific surface area and abundant electrostatic adsorption sites for the adsorption of dye molecules. In this structure, CNTs can also play the role of “threading” by interpenetrating different GO layers, so the multiple GO layers can interpenetrate and combine with each other, making them more firmly connected with the AC matrix and adjacent GO layers. It can also prevent the composite from being damaged by external force, acid and alkali treatment, extreme temperature, and adsorption desorption processes, hence, improving the cyclic adsorption stability of the GO-CNT/AC composites.

### 3.2. Crystal Structure Analysis of the GO-CNT/AC Composites

**Figure 2:**
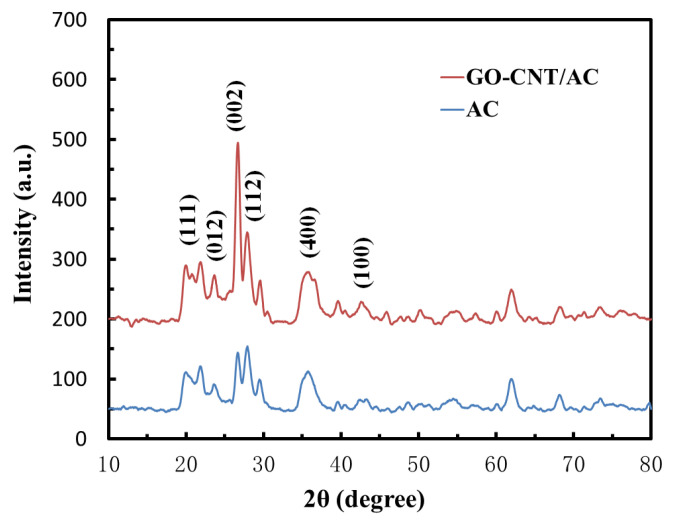
XRD spectra of AC and GO-CNT/AC composites.

The crystal structure of AC and GO-CNT/AC composites was studied by X-ray diffractometer with a wavelength of 0.154 nm. The XRD spectra of the AC and GO-CNT/AC composites are shown in Figure 2, which reveal the characteristic peaks at 2θ = 20.7°, 23.6°, 26.1°, 27.9°, 35.8°, and 42.4°, corresponding to (111), (012), (002), (112), (400), and (100), respectively [33]. Especially, compared to raw AC, after combined with the CNTs and GO, the (002) peak of the composites at 2θ = 26.1° is strongly enhanced. This is because the (002) crystal plane of CNTs also produces a strong diffraction peak at the same position, thus obviously increase the intensity of this peak. In addition, the average crystallite size (L_a_, L_c_) and the graphitization degree (g) of the composites can be calculated using the following formula [34]:

L_a_ = 1.8λ/β_100_Cosθ_100_ (3)

L_c_ = 0.9λ/β_002_Cosθ_002_ (4)

d_002_ = λ/2sinθ_002_ (5)

g = (3.440 − d_002_)/(3.440 − 3.354)·100% (6)

where L_a_ is the average crystalline width, L_c_ is the average crystalline height, λ is the wavelength of X-ray, β is the half-width, θ is the diffraction angle, d is the interplanar spacing of the crystal, and g is the graphitization degree of the samples.

Based on the above equations and the XRD spectra, the average crystallite size and the graphitization degree of AC and GO-CNT/AC composites were calculated, as listed in Table 1.

It can be seen that the calculated crystalline parameters of the GO-CNT/AC composites are larger than those of AC. This is because the XRD analysis provides a macro average result. After the addition of CNTs, which has good crystallinity, it can obviously increase the height of the (002) peak and reduce its half-width. Therefore, when calculated using Formulas (3)–(6), the macro average results show small increase in crystalline size and graphitization degree.

### 3.3. Raman Analysis of the GO-CNT/AC Composites

**Figure 3:**
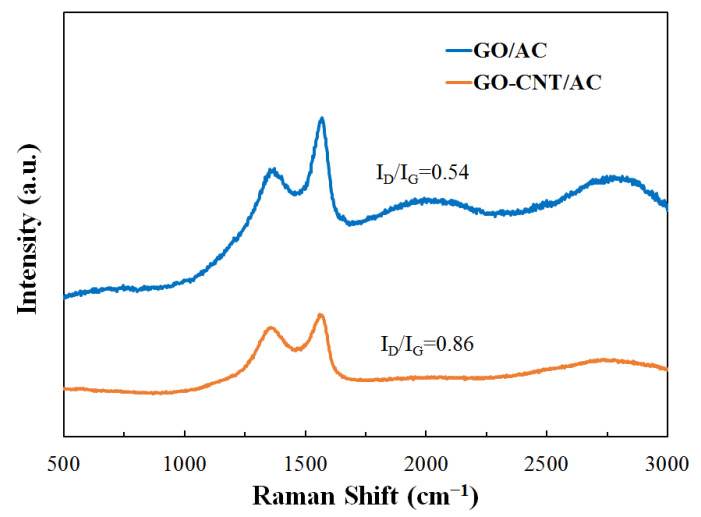
Raman spectra of GO/AC and GO-CNT/AC composites.

In order to analyze the microstructure and defects of the composites, Raman spectra were measured, as shown in Figure 3. The spectra contain two characteristic peaks near 1348 cm^−1^ and 1590 cm^−1^, which are the D-band and G-band respectively. When CNTs are added, the ratio of the peak strength (R = I_D_/I_G_) increases from 0.54 to 0.86, which correspond to the GO/AC and GO-CNT/AC composites, respectively. This is because the oxygen-containing functional groups produced by the pretreatment of CNTs increase the lattice defects and decrease the overall order of the composites. Interactions are generated between CNTs and AC surface, CNTs and GO sheets, as well as CNTs between each other. These components are linked through van der Waals force, making the composite more closely combined. A weaker 2D band appears at 2700 cm^−1^ in the spectrum of the GO/AC composite, indicating the existence of multilayer GO sheets. On the contrary, the GO-CNT/AC composite has no obvious 2D band at approximately 2700 cm^−1^, because the GO sheets form a fluffy three-dimensional structure with CNTs supported on the surface, hence improving the overall specific surface area. In addition, CNTs interlace and combine with GO sheets through “needle threading”, forming GO sheets of larger scale and strengthening the combination of the composites, thereby increasing the stability of the adsorption cycle.

### 3.4. XPS Analysis of the GO-CNT/AC Composites

**Figure 4:**
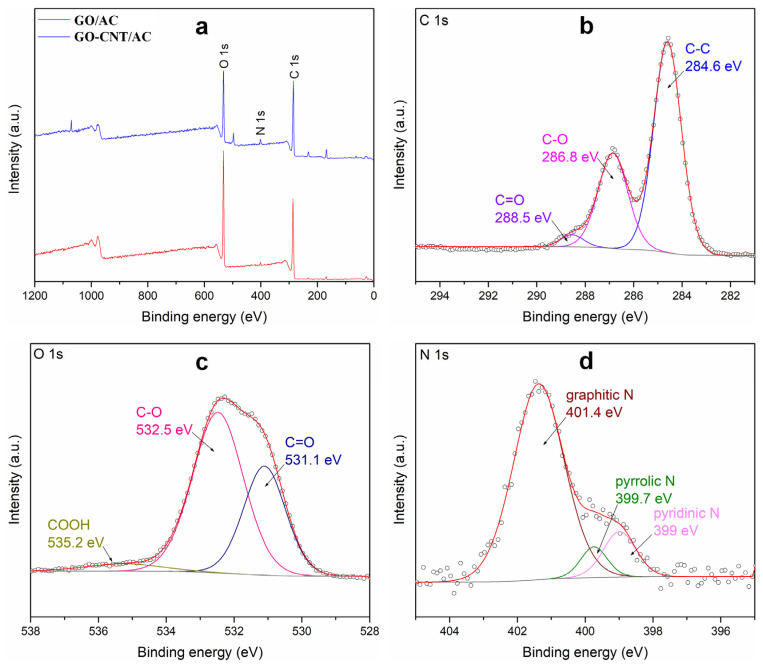
XPS full spectrum of GO/AC and GO-CNT/AC composite (**a**), and the fine spectrum of C 1s (**b**), O 1s (**c**), N 1s (**d**) in GO-CNT/AC composites.

XPS analyses were conducted to study the internal bonding of the composites, the spectra are shown in Figure 4. It can be found in Figure 4a that the full spectra of the GO/AC and GO-CNT/AC composites reveal obvious C 1s and O 1s peaks. In addition, with the addition of CNTs, GO-CNT/AC composites have obvious N 1s peaks. This is because nitric acid was used in the pretreatment of CNTs, which led to the formation of nitrogen-containing functional groups on the surface of GO-CNT/AC composites. With the CNTs attached to the surface of AC and GO, the content of nitrogen on the surface increased. Figure 4b shows the C 1s fine spectrum of the GO-CNT/AC composite. The peaks with binding energy of 284.6 eV, 286.8 eV, and 288.5 eV correspond to C-C, C-O, and C=O, respectively. The O 1s fine spectrum Figure 4c shows the peaks with the binding energy of 531.1 eV, 532.5 eV, and 535.2 eV, which correspond to C=O, C-O, and carboxyl groups, respectively. Figure 4d reveals the N 1s fine spectrum with binding energy of 399 eV, 399.7 eV, and 401.4 eV, which correspond to pyridine nitrogen, pyrrolin nitrogen, and graphite nitrogen, respectively. The types of carbon and oxygen containing bonds of the two materials have not increased significantly, indicating that the two materials are mainly constructed from physical combination by van der Waals force.

### 3.5. TG Analysis of the GO-CNT/AC Composites

**Figure 5:**
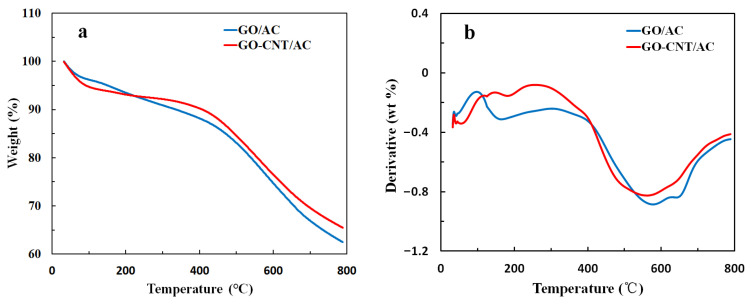
TG (**a**) and DTG (**b**) curves of GO/AC and GO-CNT/AC composites.

The thermal stability analysis of GO/AC and GO-CNT/AC composites was determined in a N_2_ atmosphere at a heating rate of 10 °C/min from 30 °C to 800 °C. The TG and DTG curves are shown in Figure 5. The weight loss ratios of the GO/AC and GO-CNT/AC composites are 37.6% and 34.5%, respectively. The weight loss of the GO-CNT/AC composites can be divided into three stages. Below 200 °C, it is mainly due to the thermal decomposition of the oxygen-containing functional groups on the surface of CNTs and GO. The loss rate in this stage peaked at 181.9 °C. At the temperature range of 200–400 °C, it is mainly due to the slow decomposition of the carbon material skeleton, with a lower loss rate. It can be seen from Figure 5b that a large amount of weight loss occurred between 400 and 700 °C. At this stage, the test temperature gradually exceeds the preparation temperature of AC, with the discharge of hetero-atoms and the rearrangement of carbon atoms in the carbon matrix. The original crystal structure disintegrates, and some of the components are taken away by the carrier gas, resulting in the reduction of the sample mass. The weight loss rate reaches the maximum at 562.2 °C, and then gradually decreases with the increasing temperature. However, the weight loss is still in progress at 800 °C, which indicates that the phase structure of the composites still has not reached stability. Furthermore, combined with the XRD testing results in Table 1, both the AC and GO-CNT/AC composites have low graphitization degree and small crystalline size, which also reduces their stability at high temperature. The weight loss of GO-CNT/AC composites at 200~800 °C is 27.1% of the total weight, smaller than that of GO/AC composites, which is 30.2%. According to the results of the TG analysis, CNTs improve the thermal stability of the composites, which expands the application fields of GO-CNT/AC composites for adsorption at higher temperatures.

### 3.6. N_2_ Adsorption–Desorption Analysis of the GO-CNT/AC Composites

**Figure 6:**
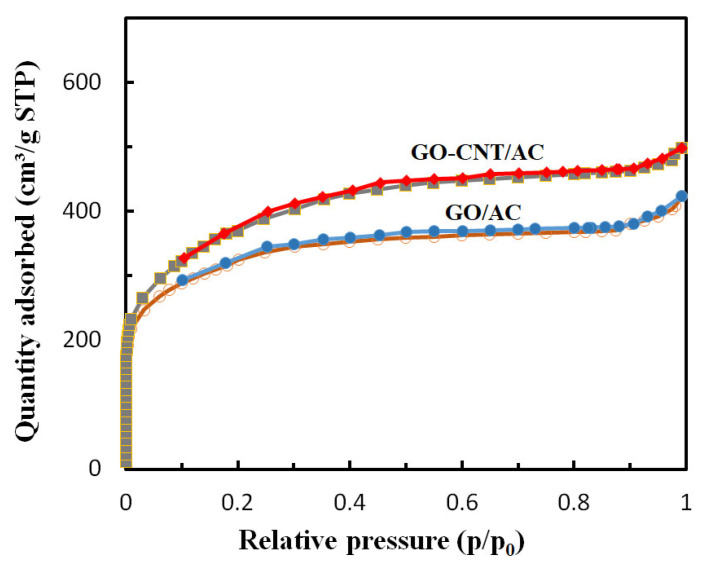
N_2_ adsorption–desorption isotherms of the GO/AC and GO-CNT/AC composites.

The N_2_ adsorption–desorption test was carried out to analyze the specific surface area and pore structure of the GO/AC and GO-CNT/AC composites, with the N_2_ adsorption–desorption isotherms shown in Figure 6. The calculated specific surface area and pore structure parameters are listed in Table 2. Before the addition of CNTs, the specific surface area and total pore volume of the GO/AC composites are 1104.93 m^2^/g and 0.87 m^3^/g, respectively. When CNTs are added to the system, the specific surface area and total pore volume of the GO-CNT/AC composites increase to 1361.86 m^2^/g and 1.03 m^3^/g, and increased by 23.3% and 18.4%, respectively. The testing results reveal that the rich nanoscale pores and defects of CNTs, the fold gaps and nanoholes of the GO sheet, and the gaps between CNTs, GO, and AC not only increase the specific surface area and pore volume, but also significantly increase the active adsorption sites of the composites, which is ultimately beneficial to the improvement of its adsorption performance.

### 3.7. Adsorption Properties of GO-CNT/AC Composites

#### 3.7.1. Influence of the CNT Concentration on the Adsorption Properties

**Figure 7:**
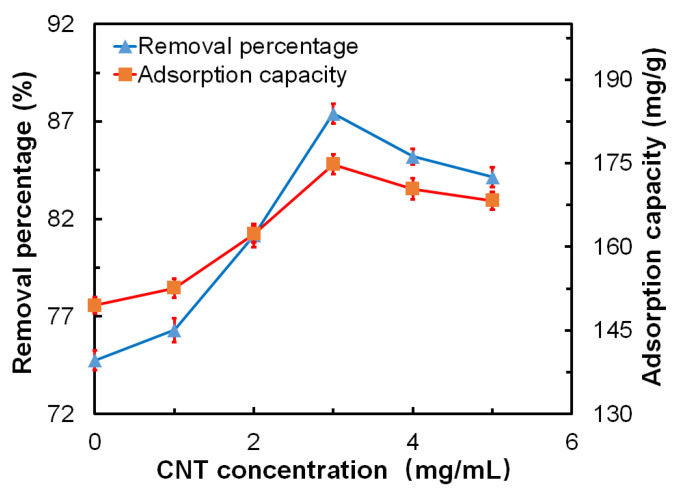
Influence of the CNT concentration on the removal percentage and adsorption capacity of the GO-CNT/AC composites [Conditions: adsorbent amount = 100 mg, MB solution amount = 100 mL, MB solution concentration = 200 mg/L, pH = 7, adsorption time = 300 min, CNT concentrations were 0, 1, 2, 3, 4 and 5 mg/mL, respectively].

The GO-CNT/AC composites were synthesized with different CNT concentrations, and then, 100 mg of GO-CNT/AC composites were immersed into 100 mL of MB solutions with a concentration of 200 mg/L, pH value of 7, and absorption time of 300 min for the adsorption investigation. To make a comparison, the adsorption property of raw AC is also tested under the same conditions. The influence of the CNT concentration on the removal percentage and adsorption capacity of the composites was investigated and shown in Figure 7. The CNT concentration referred to the content of CNTs in the GO-CNT solution which was used to prepare GO-CNT/AC composites. The CNT concentrations were set as 0, 1, 2, 3, 4, and 5 mg/mL, respectively, as described in Section 2.4. In this study, when the CNT concentration increases from 0 mg/mL to 3 mg/mL, the MB removal percentage of GO-CNT/AC composites increases from 74.7% to 87.4%, and the MB adsorption capacity of GO-CNT/AC composites increases from 149.5 mg/g to 174.8 mg/g. Compared with the raw AC material, the removal percentage is 55.2% and the adsorption capacity is 109.8 mg/g, which are increased by 58.3% and 59.2%, respectively. When the CNT concentration is higher than 3 mg/mL, both the removal percentage and the adsorption capacity of the composites start to decrease. The reason is that at relatively lower CNT concentration, the GO sheets are linked to the surface of AC through the CNT “spring”, which plays a supporting role in exposing the surface of the GO sheet, and further increasing the specific surface area of the whole system. Moreover, the above composites contain a large number of negatively charged adsorption sites and micropores, which greatly increase the adsorption performance of the composites mainly based on electrostatic adsorption and microporous adsorption. When the CNT concentration is too high, it tends to agglomerate between the GO sheets and on the AC surfaces, which can further block the pores of the AC matrix, thus reducing the adsorption property of the GO-CNT/AC composites.

#### 3.7.2. Influence of the Adsorbent Amount on the Adsorption Properties

**Figure 8:**
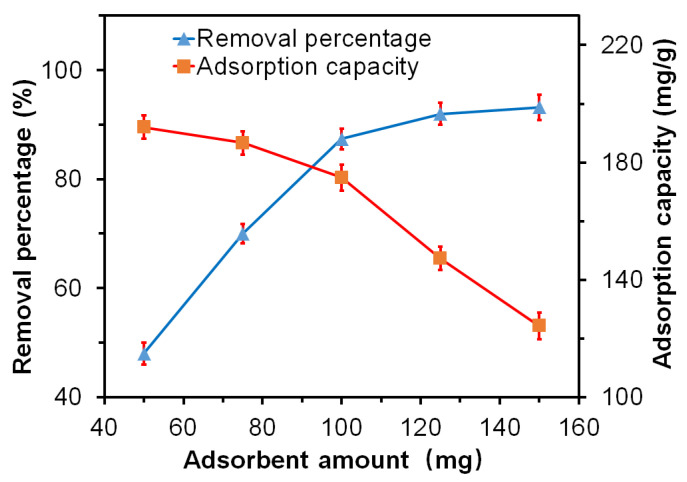
Influence of the adsorbent amount on the removal percentage and adsorption capacity of the GO-CNT/AC composites [Conditions: MB solution amount = 100 mL, MB solution concentration = 200 mg/L, pH = 7, adsorption time = 300 min, CNT concentration = 3 mg/mL, adsorbent amounts were 50, 75, 100, 125 and 150 mg, respectively].

To investigate the influence of the adsorbent amount on the adsorption properties of the GO-CNT/AC composites, different amounts of composites were placed in 100 mL of MB solutions with a concentration of 200 mg/L for the adsorption test, the pH value of 7, absorption time of 300 min, CNT concentration of 3 mg/mL, and adsorbent amounts of 50, 75, 100, 125 and 150 mg respectively were adopted. The removal percentage and adsorption capacity of the samples were calculated subsequently, as shown in Figure 8. When the adsorbent amount increases from 50 mg to 150 mg, the removal percentage increases from 48.03% to 93.16%; the adsorption capacity decreases from 192.12 mg/g to 124.21 mg/g. The reason is that when the adsorbent amount increases, the total surface area provided by the composite increases, and the total amount of active adsorption sites also increases significantly. Hence, more MB molecules can be adsorbed from the solution, resulting in an obvious increase in the removal percentage. On the other hand, as shown in Formula (2), the adsorption capacity was defined as the ratio of the total amount of adsorbed MB molecules to the adsorbent mass. When the adsorbent amount increases from 50 mg to 150 mg, although more MB molecules are adsorbed, the increasing amount of the adsorbent is lager, which leads to a decrease in the adsorption capacity with increasing adsorbent amount.

#### 3.7.3. Influence of the MB Concentration on the Adsorption Properties

**Figure 9:**
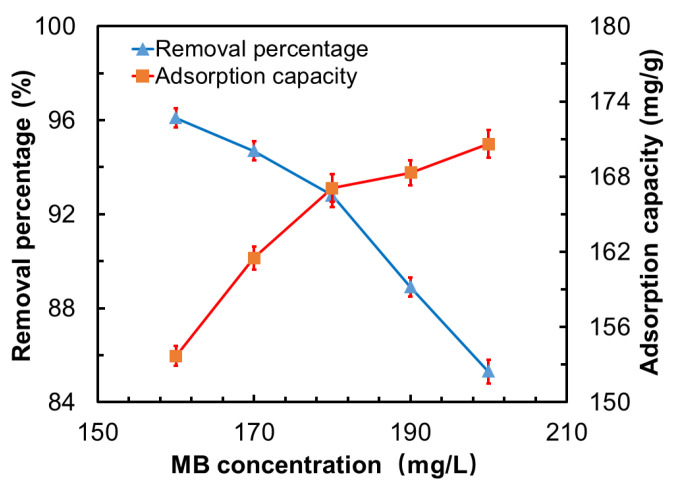
Influence of the MB concentration on the removal percentage and adsorption capacity of the GO-CNT/AC composites [Conditions: adsorbent amount = 100 mg, MB solution amount = 100 mL, pH = 7, adsorption time = 300 min, CNT concentration = 3 mg/mL, MB solution concentrations were 160, 170, 180, 190, and 200 mg/L, respectively].

To investigate the influence of the MB concentration on the adsorption properties of the GO-CNT/AC composites, 100 mg of composite was placed in 100 mL of MB solutions with different initial concentrations (160–200 mg/L) for the adsorption test, the pH value of 7, absorption time of 300 min, and CNT concentration of 3 mg/mL were adopted, and the results are shown in Figure 9. When the initial MB concentration increases from 160 mg/g to 240 mg/g, the removal percentage of the composites decreases from 96.1% to 85.3%, and the adsorption capacity increases from 153.7 mg/g to 170.6 mg/g. The reason is that when the initial MB concentration increases, the number of MB molecules near the surface of the absorbent increases significantly, and the surface active sites of GO-CNT/AC composites are more fully surrounded by dye molecules, hence, more dye molecules are captured and absorbed by the composites, which leads to the increasing of the adsorption capacity [35,36]. However, as an adsorbent relying on micropores and electrostatic adsorption sites, GO-CNT/AC composite has a limited adsorption capacity. When the saturated adsorption state is reached, the removal percentage will decrease if the initial MB concentration continues to increase.

#### 3.7.4. Influence of the pH Value on the Adsorption Properties

**Figure 10:**
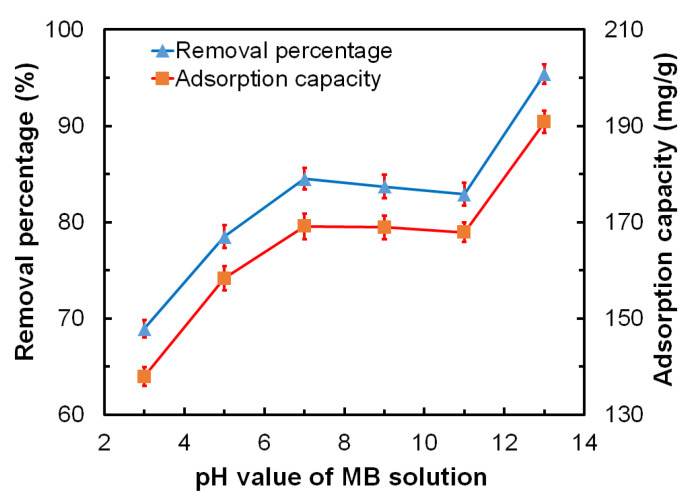
Influence of the pH value on the removal percentage and adsorption capacity of the GO-CNT/AC composites [Conditions: adsorbent amount = 100 mg, MB solution amount = 100 mL, MB solution concentration = 200 mg/L, adsorption time = 300 min, CNT concentration = 3 mg/mL, pH values were 3, 5, 7, 9, 11 and 13, respectively].

To evaluate the influence of the pH value on the adsorption performance of the GO-CNT/AC composites, 100 mg of GO-CNT/AC composites was placed in 100 mL of MB solutions with a concentration of 200 mg/L, absorption time of 300 min, CNT concentration of 3 mg/mL, and pH values of 3, 5, 7, 9, 11, and 13, respectively. The removal percentage and adsorption capacity were calculated, which are shown in Figure 10. With the increasing pH value, both the removal percentage and the adsorption capacity of the composites show an increasing trend. When the pH value increases from 3 to 13, the removal percentage increases from 68.9% to 95.4%, and the adsorption capacity increases from 137.9 mg/g to 190.8 mg/g. In this process, the removal percentage and the adsorption capacity of GO-CNT/AC composites are increased by 38.5% and 39.6%, respectively, indicating that the pH value of the MB solution has a significant impact on the adsorption performance of the composites. This is because that as a cationic dye, MB molecules are positively charged in the solution. When the pH value of the solution is less than 7, there are a large number of positively charged H^+^ ions in the acidic system. These H^+^ ions adsorb on the surface of the absorbent and generate electrostatic repulsion with MB molecules [37,38,39], thus restraining the diffusion and adsorption of MB molecules on the surface of the absorbent, and ultimately reducing the adsorption capacity of the composites. When the pH is higher than 7, there are a large number of OH^-^ ions in the solution, which adsorb on the surface of the absorbent and further combine with MB molecules through the electrostatic attraction effect [38,39]. This is conducive to the diffusion and adsorption of MB molecules on the surface of the composite, and also promotes MB molecules to enter the microporous structure of the composite, further improving the adsorption effect of the composites. Finally, the adsorption capacity of the GO-CNT/AC composite was significantly increased. However, it can be found that with the increasing pH value, the adsorption capacity of the composites has a slightly downward trend when the pH value is between 7 and 11. This is because the generation of hydrophilic functional groups on the CNT surface, which weakens the stable link between AC and GO, leads to partial disintegration of the composites. Therefore, the electrostatic adsorption sites on the surface of GO-CNT/AC composites decrease, which causes a decreasing adsorption capacity. However, when the alkalinity is further enhanced to strong alkalinity (such as pH = 13), a large number of OH^-^ ions are ionized, and the remaining AC, CNTs, and GO can generate more electrostatic adsorption sites, which promotes the adsorption process. Therefore, the adsorption properties of the composites are further improved at this time.

In summary, when the pH value of the MB solution is 13, the CNT concentration is 3 mg/mL, and the MB concentration is 200 mg/L, the adsorption property of the composite is the best, with an adsorption capacity of 190.8 mg/g and a removal percentage of 95.4%. Compared with the raw AC, the adsorption capacity and removal percentage of the composites are increased by 73.8% and 72.8%, respectively.

#### 3.7.5. Cyclic Adsorption Properties of the GO-CNT/AC Composites

**Figure 11:**
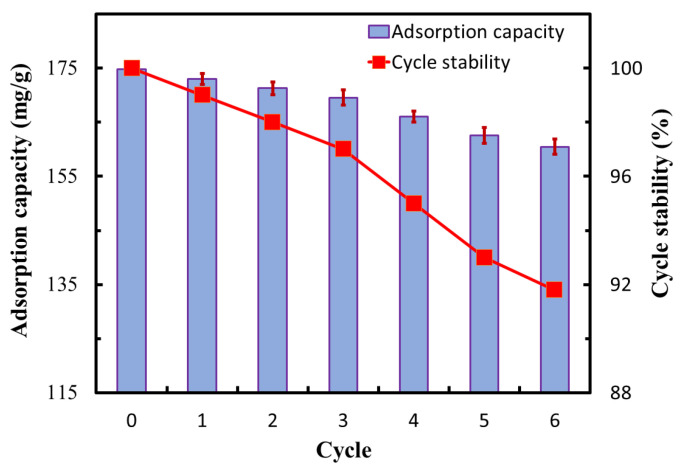
The adsorption capacity and cycle stability of the GO-CNT/AC composites [Conditions: adsorbent amount = 100 mg, MB solution amount = 100 mL, MB solution concentration = 200 mg/L, pH = 7, adsorption time = 300 min, CNT concentration = 3 mg/mL].

To investigate the cyclic adsorption properties of the GO-CNT/AC composites, six cycles of adsorption–desorption tests were conducted. The adsorbent amount of 100 mg, MB solution amount of 100 mL, MB solution concentration of 200 mg/L, pH value of 7, adsorption time of 300 min, and CNT concentration of 3 mg/mL were applied. The adsorption capacity after each cycle was measured, as shown in Figure 11. The adsorption capacity of the GO-CNT/AC composites for MB in the initial cycle is 174.8 mg/g. After six cycles, the adsorption capacity is 160.5 mg/g, with a cycle stability (C_s_ = Q_t_/Q_1_) of 91.8%. The high stability of the adsorption–desorption cycle of the composites reveals that the addition of CNTs creates a stable link between the AC and GO sheets. During the adsorption–desorption cycle, the fluffy structure created by CNTs is easier to adsorb and desorb dye molecules. In the last two cycles, the loss rate of the cycle stability is slightly higher because the desorption process requires acid treatment. Thus, the original stable link in the GO-CNT/AC composite is destroyed, which causes the gradual disintegration of GO-CNT/AC composite, reducing the cycle stability of the system. However, even after six cycles of testing, the adsorption capacity of the composites is still high, indicating that the GO-CNT/AC composites still maintain structural integrity, the GO and CNTs are still stably linked with AC. Thus, the addition of CNTs can improve both the adsorption capacity and the cycle stability of the GO-CNT/AC composites.

### 3.8. Adsorption Kinetics Study of the GO-CNT/AC Composites

In order to further investigate the adsorption process of GO-CNT/AC composites on MB solution, the pseudo-first-order (PFO) kinetic models, pseudo-second-order (PSO) kinetic models, and intra-particle diffusion (IPD) kinetic models are adopted to analyze the kinetics of the adsorption processes. The PFO, PSO, and IPD kinetic model can be described by the following formulas [40,41,42,43].

(7) $$\ln\left(Q_{e}-Q_{t}\right)=\ln Q_{e}-k_{1}t$$

(8) $$\frac{t}{Q_{t}}=\frac{1}{k_{2}Q_{e}{}^{2}}+\frac{t}{Q_{e}}$$

(9) $$Q_{t}^{}=k_{p}t^{1/2}+C_{p}$$

where Q_e_ is the equilibrium adsorption capacity (mg/g), Q_t_ is the adsorption capacity at time t (mg/g), k_1_ is the PFO kinetic constant (/min), k_2_ is the PSO kinetic constant (g/(mg·min)), K_p_ is the IPD kinetic constant (mg/(g·min^1/2^)), C_p_ is the intercept (mg/g).

**Figure 12:**
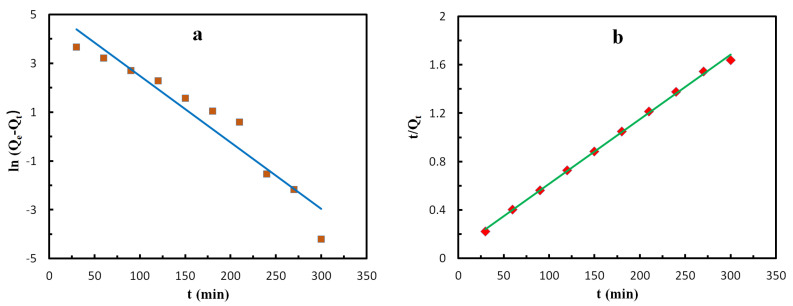
Fitting plots of the PFO (**a**) and PSO (**b**) models in the MB adsorption process.

GO-CNT/AC composites (100 mg) were immersed in MB solution (100 mL) with a concentration of 200 mg/L, and the adsorption capacity of the samples was recorded every 30 min. Then, plots based on the PFO model (ln(Q_e_ − Q_t_) − t) and PSO model (t/Q_e_ − t) were drawn and fitted, as shown in Figure 12. Table 3 shows the kinetic parameters of the adsorption process calculated from the slope and intercept of the fitting curve. It can be seen from the table that the correlation coefficient R^2^ of the PFO kinetics fitting model is 0.92, and the correlation coefficient R^2^ of the PSO kinetics fitting model is 0.99. Obviously, the PSO kinetics model is more suitable for fitting the actual adsorption process of the GO-CNT/AC composites. Compared with the theoretical value of adsorption capacity, the actual adsorption capacity is lower, indicating that the adsorption performance of the GO-CNT/AC composites still has the potential for further improvement. Meanwhile, due to the addition of CNTs into the system, the adsorption structure changes from a plane to a 3D network structure. Simple PFO and PSO kinetic models can no longer perfectly describe the adsorption kinetics of GO/CNT/AC composites, so the IPD kinetic model is needed to further analyze the adsorption process.

**Figure 13:**
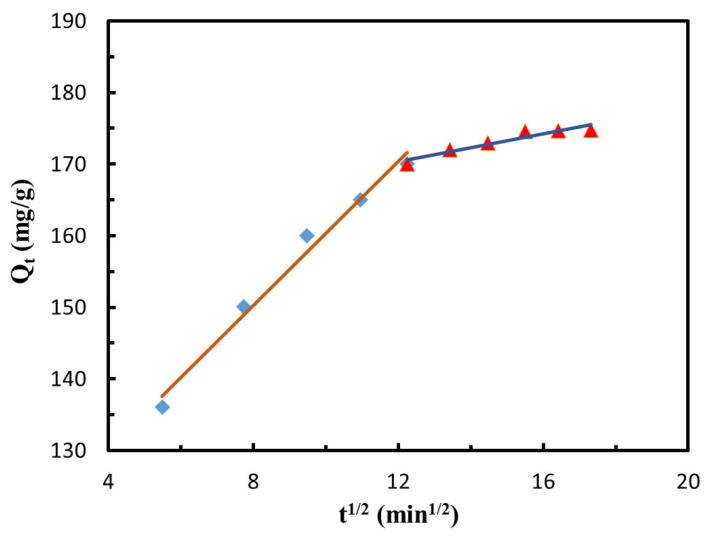
Fitting diagram of the IPD model in the MB adsorption process.

Figure 13 shows the fitting results of the IPD model describing the adsorption process of GO-CNT/AC composites for MB solution. The calculated kinetic constants are shown in Table 4. The IPD model of the adsorption process can be divided into two stages. In the first stage, MB molecules diffuse on the outer surface of the GO-CNT/AC composites. This process is mainly realized by physical adsorption, including microporous adsorption and electrostatic adsorption on the surface of the composites. In this stage, the diffusion coefficient k_p1_ is 5.02 mg/(g·min^1/2^). The diffusion and adsorption processes were carried out simultaneously and the adsorption speed was fast. It can be observed that MB solution becomes shallow rapidly. In the second stage, the diffusion coefficient k_p2_ is 0.97 mg/(g·min^1/2^), which is significantly lower than that in the first stage. In this stage, MB molecules gradually diffuse into the AC pores, GO folds, and CNT walls in the GO-CNT/AC composite. The second stage has a lower diffusion rate because the concentration of MB molecules in the external solution decreases significantly, it cannot fully contact the active adsorption sites of the GO-CNT/AC composites, resulting in a slower adsorption rate, and the adsorption process gradually transitions to the equilibrium state.

## 4. Conclusions

In this study, GO-CNT/AC composites are prepared from the combination of GO, CNTs, and AC by solution impregnation and freeze-drying methods, constructing three-dimensional structures with nano-micro hierarchical pores, with highly efficient adsorption properties suitable for the removal of wastewater pollutants. The morphology, microstructure, chemical properties, adsorption performance, and adsorption kinetics are systematically investigated. The results show that GO and CNTs are uniformly dispersed on the surface of AC, significantly restrain the agglomeration phenomenon. The added CNTs played a “spring” role, supporting the gap between the GO sheets and AC. Meanwhile, stable links are formed between GO, CNTs, and AC, realizing the optimal matching of the microstructure. The specific surface area of the GO-CNT/AC composite is 1361.86 m^2^/g, and its adsorption capacity is significantly improved. The influences of the CNT contents, adsorbent amounts, MB solution concentrations, and pH values on the adsorption properties of GO-CNT/AC composites are analyzed. With the increasing CNT concentration, the adsorption capacity of GO-CNT/AC composites increases from 149.5 mg/g to 174.8 mg/g, and the removal percentage increases from 74.7% to 87.4%. With the increasing initial MB concentration, the adsorption capacity of the composites increases from 153.7 mg/g to 170.6 mg/g, and the removal percentage decreases from 96.1% to 85.3%. When the pH value of the MB solution is 13, the CNT concentration is 3 mg/mL and the MB concentration is 200 mg/L, the adsorption property of the composite is the best, with an adsorption capacity of 190.8 mg/g and a removal percentage of 95.4%. Compared with the raw AC, the adsorption capacity and removal percentage of the composites are increased by 73.8% and 72.8%, respectively. The GO-CNT/AC composite has good cyclic adsorption performance, with a cyclic stability of 91.8% after six rounds of MB adsorption–desorption cycles. The kinetic analysis shows that the adsorption process conforms to the PSO kinetic model. By fitting of the IPD model, the adsorption mechanisms of the GO-CNT/AC composites to MB are divided into two adsorption stages and described respectively.

## Figures and Tables

**Table 1:** Average crystallite size and graphitization degree of AC and GO-CNT/AC composites.

Samples	La (nm)	Lc (nm)	g (%)
AC	15.49	10.09	24
GO-CNT/AC composites	16.71	11.35	27

**Table 2:** Specific surface area and pore structure parameters of the composites.

Samples	S_BET_ (m^2^/g)	S_mic_ (m^2^/g)	V_tot_ (cm^3^/g)	V_mic_ (cm^3^/g)
GO/AC	1104.93	793.69	0.87	0.59
GO-CNT/AC	1361.86	936.68	1.03	0.76

Note: S_BET_ is the specific surface area, S_mic_ is the micropore surface area, V_tot_ is the total pore volume, V_mic_ is the micropore volume.

**Table 3:** Calculated kinetic constants of the PFO and PSO models in the MB adsorption process.

Experimental Adsorption Capacity	Pseudo-First-Order			Pseudo-Second-Order		
Actual value Q_e_ (mg/g)	Theoretical value Q_e_ (mg/g)	k_1_ (/min)	R^2^	Theoretical value Q_e_ (mg/g)	k_2_ (g/(mg·min))	R^2^
174.81	184.75	0.027	0.92	188.67	3.6 × 10^−4^	0.99

**Table 4:** Calculated kinetic constants of the IPD model in the MB adsorption process.

Initial MB Concentration	Intra-Particle Diffusion					
C_0_ (mg/L)	k_p1_ (mg/(g·min^1/2^))	C_p1_ (mg/g)	R^2^	k_p2_ (mg/(g·min^1/2^))	C_p2_ (mg/g)	R^2^
200	5.02	110.06	0.98	0.97	158.79	0.91

## Data Availability

The raw data presented in this study are available on request from the corresponding author.
